# Safety of sildenafil in premature infants with severe bronchopulmonary dysplasia (SILDI-SAFE): a multicenter, randomized, placebo-controlled, sequential dose-escalating, double-masked, safety study

**DOI:** 10.1186/s12887-020-02453-7

**Published:** 2020-12-14

**Authors:** Simone Schneider, Mary Bailey, Tracy Spears, Charles R. Esther, Matthew M. Laughon, Christoph P. Hornik, Wesley Jackson

**Affiliations:** 1 Department of Pediatrics, School of Medicine, The University of North Carolina at Chapel Hill, Chapel Hill, NC USA; 2 Duke Clinical Research Institute, Durham, NC USA; 3 Department of Pediatrics, Duke University School of Medicine, Durham, NC USA

**Keywords:** Pulmonary hypertension, Premature infant, Bronchopulmonary dysplasia, Randomized control trial, Sildenafil, Hypotension, Mean arterial pressure, Echocardiogram

## Abstract

**Background:**

Pulmonary hypertension is a deadly complication of bronchopulmonary dysplasia, the most common pulmonary morbidity of prematurity. Despite these catastrophic consequences, no evidence-based therapies are available for the prevention of pulmonary hypertension in this population. Sildenafil is a potent pulmonary vasodilator approved by the US Food and Drug Administration for the treatment of pulmonary hypertension in adults. Preclinical models suggest a beneficial effect of sildenafil on premature lungs through improved alveolarization and preserved vascular development. Sildenafil may therefore prevent the development of pulmonary hypertension associated with lung disease of prematurity by reducing pulmonary vascular remodeling and lowering pulmonary vascular resistance; however, clinical trial evidence is needed. The present study, supported by the National Institutes of Health’s National Heart Lung and Blood Institute, will generate safety, pharmacokinetics, and preliminary effectiveness data on sildenafil in a population of premature infants with severe bronchopulmonary dysplasia at risk for pulmonary hypertension.

**Methods:**

We have designed a multicenter, randomized, placebo-controlled, sequential dose-escalating, double-masked, safety trial of sildenafil in premature infants with severe bronchopulmonary dysplasia. We will randomize 120 premature infants < 29 weeks gestational age with severe bronchopulmonary dysplasia at 32–40 weeks postmenstrual age in a dose-escalating approach 3:1 (sildenafil: placebo) sequentially into each of 3 cohorts at ~ 30 clinical sites. Participants will receive up to 34 days of study drug, followed by 28 days of safety monitoring. The primary outcome will be safety as determined by incidence of hypotension. Secondary outcomes will include pharmacokinetics and preliminary effectiveness of sildenafil based on presence or absence of pulmonary hypertension diagnosed by echocardiography at the end of treatment period.

**Discussion:**

Sildenafil is a promising intervention to prevent the development of pulmonary hypertension in premature infants with bronchopulmonary dysplasia. Clinical trials of sildenafil specifically designed for premature infants are urgently needed. The current study will make substantial contributions to scientific knowledge of the safety of sildenafil in premature infants at risk for pulmonary hypertension. Results from the study will be used by investigators to inform the design of a pivotal efficacy trial.

**Trial registration:**

ClinicalTrials.govNCT04447989. Registered 25 June 2020.

**Supplementary Information:**

The online version contains supplementary material available at 10.1186/s12887-020-02453-7.

## Background

Each year in the United States, over 50,000 infants are born < 29 weeks gestational age (GA), of whom nearly 40% develop bronchopulmonary dysplasia (BPD) [1]. The severity of BPD is defined by the level of respiratory support at 36 weeks postmenstrual age (PMA) [2]. Premature infants with severe BPD are particularly challenging to manage and often suffer from multiple complications and comorbidities, including prolonged hospitalization, need for home respiratory support, and death [3–5]. A common comorbidity in infants with severe BPD is pulmonary hypertension (PH). Infants with BPD-associated PH have significantly higher mortality compared to infants with BPD who do not develop PH [3–5].

The temporal relationship between BPD and PH in premature infants is complex. Approximately 20% of infants with severe BPD develop PH, and nearly half of these infants die, most often due to right-sided heart failure [4, 6–9]. Early PH in the first post-natal week is a risk factor for increased BPD severity [6]. However, most infants at risk for BPD develop PH later in life: one prospective study of extremely low birth weight infants found that 65% of infants with BPD-related PH had no evidence of PH on screening echocardiograms obtained at 4–6 weeks of life [10].

There are currently no US Food and Drug Administration (FDA) approved or evidence-based drugs to prevent or treat PH in premature infants with BPD. Sildenafil is a potent inhibitor of phosphodiesterase type 5, the predominant isoform in the lung, which metabolizes cyclic guanosine monophosphate and produces pulmonary vasodilation by potentiating the effects of endogenous nitric oxide. The FDA has approved the use of sildenafil for treatment of pulmonary arterial hypertension in adults to improve exercise ability and delay clinical worsening [11]. The FDA does not recommend sildenafil for PH in children 1 to 17 years of age and there are no recommendations regarding its use in neonates and infants.

The objectives of this trial are to characterize the safety and pharmacokinetics (PK) of sildenafil in premature infants with severe BPD, as well as provide data on efficacy for the prevention of PH in this population. Funding for this study is provided by the National Institutes of Health’s National Heart Lung and Blood Institute (NHLBI; R61HL147833).

## Methods/design

This is a multicenter, randomized, placebo-controlled, sequential dose-escalating, double masked, safety study of sildenafil in premature infants with severe BPD. The primary objective of the study is to describe the safety of sildenafil as determined by incidence of hypotension. We will analyze the PK and preliminary effectiveness of sildenafil in the prevention of PH in premature infants with severe BPD as secondary objectives.

### Study subjects

Premature infants in the Neonatal Intensive Care Unit (NICU) will be randomized into 3 cohorts with a ratio of 3:1 (sildenafil:placebo). Each sequential cohort will receive a higher dose of sildenafil for those randomized to the treatment arm (Table 1). There will be approximately 40 randomized and dosed participants in each cohort for a total of up to 120 participants.

**Table 1:** Number of Participants (N) and Dosing Scheme

Cohort^**a**^	Treatment Group		Sildenafil Dosing^**b**^ (mg/kg q 8 h)		Total (N)
	Placebo (N)	Sildenafil (N)	IV	Enteral	
1	10	30	0.5	1	40
2	10	30	1	2	40
3	10	30	2	4	40

*Abbreviations*: *IV* intravenous, *N* number of participants, *q* every

^a^Participants will be enrolled into cohorts sequentially (i.e., Cohort 1 then Cohort 2 then Cohort 3) based on safety

^b^Route of administration should be via IV route if patient has an IV and IV administration is feasible. However, route of administration, IV or enteral, is left to investigator discretion

To be eligible to participate in this study, an infant must meet all of the following inclusion and exclusion criteria:

#### Inclusion criteria

1. Documented informed consent from parent or guardian, prior to study procedures
2. < 29 weeks GA at birth
3. 32–40 weeks PMA
4. Receiving respiratory support at enrollment:
  If 32–36 weeks PMA: mechanical ventilation (high frequency or conventional)
  If > 36–40 weeks PMA: mechanical ventilation (high frequency or conventional) OR continuous positive airway pressure (CPAP)

Note:

- Criteria 3 and 4 define severe BPD for the purposes of this study.
- CPAP is defined as any of the following:
  - Nasal cannula > 2 liters per minute (LPM)
  - Nasal continuous positive airway pressure (NCPAP)
  - Nasal intermittent positive pressure ventilation (NIPPV)
  - Noninvasive neurally adjusted ventilatory assist (NAVA)
  - Any other device designed to provide positive pressure through a nasal device (e.g., RAM cannula, etc.)

#### Exclusion criteria

1. Previous enrollment and dosing in this study
2. Previous exposure to sildenafil within 7 days prior to randomization
3. Previous exposure to vasopressors within 24 h prior to randomization
4. Previous exposure to inhaled nitric oxide within 24 h prior to randomization
5. Previous exposure to milrinone within 24 h prior to randomization
6. Evidence of PH or moderate/large patent ductus arteriosus (PDA) on the most recent echocardiogram performed within 14 days prior to randomization
7. Known major congenital heart defect requiring medical or surgical intervention in the neonatal period
8. Known allergy to sildenafil
9. Known sickle cell disease
10. Aspartate aminotransferase (AST) > 225 U/L < 72 h prior to randomization
11. Alanine aminotransferase (ALT) > 150 U/L < 72 h prior to randomization
12. Any condition that would make the participant, in the opinion of the investigator, unsuitable for the study

#### Recruitment sites

Approximately 30 sites will be recruiting patients for the study. Sites were selected based on prior participation in infant trials including those conducted by the National Institute of Child Health and Human Development Pediatric Trials Network. These research teams have extensive experience interacting with and enrolling the target population. Prior to and during the enrollment period for the study, sites will participate in conference calls to discuss recruitment and retention plans, including strategies for building rapport, increasing awareness, and enrolling the study population.

Potential subjects will be identified in the NICU at participating sites. Due to the nature of the condition, a minority population is intrinsically overrepresented (minority population represents 30–40% of infants with BPD; https://www.marchofdimes.org/peristats). Therefore, the study team does not anticipate difficulty with inclusion of individuals commonly underrepresented in research studies.

#### Baseline screening

Prior to initiation of the first dose of study drug, participant and maternal demographics, mean arterial pressures (MAP), concomitant medications, laboratory evaluations (AST, ALT; when available, values for platelets, phosphorus, chloride, alkaline phosphatase, and caffeine level), and respiratory assessments will be collected (Additional file 1: Appendix Table 1). Echocardiogram reports will be collected if performed per local standard of care < 14 days prior to start of study drug. If not performed per local standard of care < 14 days prior to start of study drug, an echocardiogram will be required to confirm eligibility.

### Treatment protocol

#### Randomization

Participants will be centrally randomized via electronic data capture to maintain a 3:1 ratio within each cohort of sildenafil (*n* = 30) to placebo (*n* = 10). Participants from a multiple gestation will be randomized independently as they become eligible. Study randomization codes and replacement codes for all cohorts will be generated and uploaded prior to the enrollment of the first participant. If more than one infant from a multiple gestation is randomized, the sibling relationship between participants will be collected. Investigators are encouraged to use IV dosing if the patient has IV access. If the participant does not have an IV or if the clinical team elects to use enteral dosing, then the dose of study drug will be twice that of the IV dosing (from the product label). Placebo will be administered either enterally or IV in the same manner. Infants randomized to the placebo treatment group will receive the equivalent volume of dextrose 5%.

#### Masking procedures

Study drug and placebo will be acquired from the hospital pharmacy using commercially available preparations. The investigational pharmacist will be unmasked, and will prepare drug-appropriate sized syringes in a masked manner (e.g., amber syringe). Staff accessing participant outcomes will be masked to treatment. Drug or placebo will be administered by the bedside nurse. Intravenous doses will be administered as an infusion over 60 min followed by 30 min of flush. Enteral doses will be administered with feedings.

#### Dose escalation

Dose escalation of study drug will occur if the infant continues to receive exogenous oxygen or respiratory support (nasal cannula or positive pressure from any device) and AST < 225 U/L and ALT < 150 U/L within the 7 days prior to the last dose increase. Patients meeting these criteria will escalate their dose until goal dose is reached, and then receive 28 days of drug therapy at goal dosing prior to weaning or discontinuation of the study drug. (Table 2).

**Table 2:** Dose Escalations for Each Sequential Cohort

	Study Days (Dose Numbers)							
	1–2 (1–6)	3–4 (7–12)	5–6 (13–18)	7–8 (19–24)	9–10 (25–30)	11–12 (31–36)	13–14 (37–42)	15 + (43 +)
**Sildenafil Dose (mg/kg)**								
**Cohort 1**								
IV	0.25	0.5	0.5	0.5	0.5	0.5	0.5	0.5
Enteral	0.5	1	1	1	1	1	1	1
**Cohort 2**								
IV	0.25	0.5	0.75	1	1	1	1	1
Enteral	0.5	1	1.5	2	2	2	2	2
**Cohort 3**								
IV	0.25	0.5	0.75	1	1.5	1.75	2	2
Enteral	0.5	1	1.5	2	3	3.5	4	4

*Abbreviation*: *IV* intravenous

#### Treatment period monitoring

The treatment period will include Days 1–28 or last day of study drug if early withdrawal of study drug. During the treatment period, the following information will be collected and recorded while the participant is on study drug: all MAP values obtained 24 h after the first dose of study drug and serial MAP values around the time of study drug administration, concomitant medications, daily respiratory assessments, laboratory evaluations (AST, ALT; when available, values for platelets, phosphorus, chloride, alkaline phosphatase, and caffeine level), PK sampling, echocardiograms and cardiac catheterization reports, if performed per local standard of care, and serious adverse events (SAEs), including events of special interest.

#### Weaning

Sildenafil is commonly used at higher doses to treat PH in infants. In clinical practice, it is recommended to wean from these higher doses to prevent rebound effects that may be seen with abrupt discontinuation of sildenafil. Although sildenafil used in this study is for prevention of PH, the target doses in Cohorts 2 and 3 are similar to those used for the treatment of PH.

For Cohorts 2 and 3, weaning of study sildenafil or placebo will begin following the last study dose on Day 28 or if the dose escalates to a dose of ≥0.5 mg/kg IV or ≥ 1 mg/kg enteral and the participant is withdrawn from the study. Study drug will be weaned by 25% of the last dose every 2 days until off. Participants should complete weaning after 6 days (Table 3). Weaning will not be performed for participants in Cohort 1 as the risk of rebound effects is likely minimal for infants receiving low dose sildenafil.

**Table 3:** Weaning Schedule for Study Sildenafil or Placebo in Cohorts 2 and 3

Weaning Days	IV	Enteral
1–2	75% of last study dose	75% of last study dose
3–4	50% of last study dose	50% of last study dose
5–6	25% of last study dose	25% of last study dose
7	Discontinue	Discontinue

*Abbreviation*: *IV* intravenous

#### Follow-up period

The follow-up period will include Days 1–28 after the last study drug dose; last study drug dose may occur prior to Day 28 for those participants who withdraw from study drug early; on Day 28 for those participants who complete the full treatment period; or after last weaning dose for those participants who require weaning. The following information will be reported during the follow-up period: the lowest valid MAP value on follow-up days 1, 14, 21, and 28, daily respiratory assessments, laboratory evaluations, concomitant medications of special interest, follow-up echocardiograms, and SAEs, including events of special interest.

#### Respiratory assessments

Respiratory assessments will be recorded daily during treatment, weaning, and follow-up periods. The following information will be recorded from the medical chart: maximum daily fraction of inspired of oxygen (FiO2), highest level of ventilation type (i.e., high-frequency ventilation, conventional ventilation, non-invasive support, no support), and highest mean airway pressure, if receiving invasive mechanical ventilation.

#### Data management

All clinical report forms and laboratory reports must be reviewed by the clinical team and data entry staff, who will ensure that they are accurate and complete. The DCRI will be responsible for data management, quality review, analysis, and reporting of study data. Clinical data will be entered into an internet data entry system provided by the DCC. The data system includes password protection and internal quality checks, such as automatic range checks, to identify data that appear inconsistent, incomplete, or inaccurate.

#### Safety/adverse event policy

The Duke Clinical Research Institute (DCRI) Safety Surveillance team will be overseeing real-time significant adverse event collection, evaluation, and expedited regulatory reporting for this study. Site investigators will record all SAEs and ESIs from the time of the first study-specific procedure through 28 days after the last dose of the study drug*.* A clinician not involved in the study protocol will be appointed as a medical monitor and will review all valid SAEs and hypotension events at the time they are reported. If safety concerns are identified, the medical monitor may request a meeting of the Data and Safety Monitoring Board (DSMB) to review safety data. All hypotension and SAEs (study-drug related or not) will be followed until resolution. Regular meetings will be held throughout the study to review all safety events. An unscheduled DSMB review of safety data will be triggered if more than 3 patients in a cohort have their treatment discontinued or an infusion stopped due to the same type of event or 3 patients in a cohort have a suspected adverse reaction. Enrollment in the study will be suspended during this review. If the sponsor confirms that an SAE is related and unexpected, a MedWatch will be submitted to the FDA and the treatment assignment requires unblinding. The site investigator will provide direct access to all trial-related locations, source data/documents, clinical report forms, and reports for the purpose of monitoring and auditing by the sponsor, and inspection by local and regulatory authorities.

### Primary outcome measures

Safety of sildenafil will be determined by the incidence of hypotension experienced by the participants through 28 days post last dose of study drug. Hypotension will be defined as any clinically significant low blood pressure event deemed by the treating physician to require intervention with a fluid bolus or the initiation or escalation of inotropic, vasopressor, or systemic steroid therapy with the specific intent to raise blood pressure.

MAP values will be obtained at a minimum of 2 h, 1 h, and 15 min prior to the first dose of study drug or first dose of an escalation. For IV formulations MAP will be obtained at the start of the infusion, every 15 min during the infusion, and at the end of the infusion. MAP will also be assessed at 15 min, 30 min, hourly for 4 h, and one additional measurement prior to the subsequent dose. For enteral doses MAP will be obtained at the start of the enteral administration, then every 15 min for 90 min, then every 30 min for 60 min, then hourly for 4 h, then once in the remaining 2 h prior to the next dose. For subsequent IV or enteral daily doses, the lowest valid MAP value should be recorded.

### Secondary outcome measures

#### Pharmacokinetics

Blood samples will be collected after any dose following the completion of 7 days (168 h) of study drug administration. A population PK analysis will be performed. Using the final population PK model, empirical Bayesian estimates of clearance, volume of distribution, and half-life will be generated for each participant and used to calculate exposure metrics (e.g. area under the time concentration curve, maximum concentration).

#### Preliminary effectiveness

The presence or absence of PH will be determined using central review by blinded pediatric cardiologists of echocardiograms performed at the end of the study period. The echocardiograms will be reviewed using a standardized reading protocol for detection of PH [12]. The developed population PK model will be linked to the diagnosis of PH made by echocardiography at the end of the study period to develop a population PK/pharmacodynamics (PD) model. We will use the model to simulate the effect of variable sildenafil exposures on the probability of developing PH.

### Tertiary outcome measures

#### Global rank

A Global Rank Score (GRS) will be determined for each participant based on pre-specified endpoints (Additional file 2: Appendix Table 2). All endpoints for all participants will be ranked by severity (1 = worst). Each participant will receive a single rank based on the lowest ranking endpoint that they experienced during their participation in the study. Wilcoxon rank-sum test will be used to compare the ranks of participants administered placebo versus sildenafil, overall and by sildenafil dose.

#### Events of special interest

The patients enrolled in this trial will be initially critically ill and deemed to require prolonged NICU care. Such individuals are known to have a high morbidity and mortality rate. The events (Table 4) will not be included in the primary safety outcome, but they are clinical ESI that may relate to safety and that will be tracked and reported in the trial results.

**Table 4:** Events of Special Interest

Event	Definition
Sepsis	Positive blood culture with an organism not typically considered a contaminant
Urinary tract infection	Positive urine culture with an organism not typically considered a contaminant
Bacterial meningitis	Positive cerebrospinal fluid culture with an organism not typically considered a contaminant
Retinopathy of Prematurity (ROP)	ROP requiring treatment with laser photocoagulation or an anti-VEGF drug
Seizures	Determined by the treating provider
Abnormal hearing test results	Based on testing during primary hospitalization
Systemic arterial or deep venous thrombosis	Thrombosis requiring treatment with anticoagulation (e.g., heparin or low-molecular-weight heparin)
Direct hyperbilirubinemia	Conjugated serum bilirubin > 2.0 mg/dL
Elevated transaminases	ALT > 150 U/L or AST > 225 U/L
Endotracheal intubation	Intubation and transition from non-invasive to invasive ventilation, not due to planned surgical procedure (e.g., inguinal hernia repair or ROP treatment)

*Abbreviations*: *ROP* retinopathy of prematurity, *VEGF* vascular endothelial growth factor, *ALT* alanine aminotransferase, *AST* aspartate aminotransferase

### Statistical analyses

#### General analysis considerations

Analyses will be stratified by treatment assignment and dose groups: Cohort 1 Sildenafil, Cohort 2 Sildenafil, Cohort 3 Sildenafil, Overall Sildenafil and Overall Placebo. Patients dosed with IV or enteral formulations will be combined in analyses unless otherwise specified. Descriptive statistics will include number of observations, mean, standard deviation, median, minimum, and maximum for continuous variables or counts and percentages/proportions for categorical variables. Baseline assessment is the last non-missing value prior to first study drug administration.

#### Sample size calculations

The sample size of 30 in each dose group is sufficient to estimate hypotension incidence with sufficient precision. Table 5 provides widths for 95% Wilson confidence intervals in the dose groups of size 30 and the total sildenafil treatment cohort of 90 with different incidence rates. If hypotension occurs with an incidence rate of 0.05, it has a 78% chance of being observed at least once in a dose group and a 99% chance of being observed at least once in the total sildenafil cohort.

**Table 5:** Widths for 95% Wilson Confidence Intervals

***N*** = 30			***N*** = 90		
Rate	Width	95% CI	Rate	Width	95% CI
0.1	0.22	0.04–0.26	0.1	0.13	0.05–0.18
0.2	0.28	0.10–0.37	0.2	0.16	0.13–0.29
0.3	0.31	0.17–0.48	0.3	0.19	0.22–0.40

*Abbreviations*: *CI* confidence interval, *N* number of participants

#### Safety analysis

The primary safety endpoint is number and percent of hypotension events and will be summarized. Serious adverse events will be presented overall and by each Medical Dictionary for Regulatory Activities (MedDRA) system organ class and preferred term. Severity and relationship to sildenafil or placebo will be provided. ESI will be presented overall and summarized by participant characteristics. Prior and concomitant medications will be summarized by World Health Organization (WHO) drug class. Laboratory data, including change from baseline, will be tabulated by treatment groups.

#### PK analysis

A PK dataset will be created by the data coordinating center to include sildenafil and its primary metabolite desmethyl sildenafil (DMS) concentrations, dosing information, and clinical data for each subject enrolled in Cohorts 1, 2 and 3 of the sildenafil trial. Sildenafil and DMS PK profiles will be constructed from the observed drug concentration-time data. The dataset will also include mean arterial pressure values.

A population PK analysis will be performed using the sildenafil and DMS data to determine compartmental population PK parameters (i.e., clearance and volume) with the program NONMEM (Icon Solutions, Ellicott City, MD, USA). One and two compartment structural models will be explored for both sildenafil and DMS. Samples obtained after intravenous and enteral administration routes will be combined in a single model.

#### Preliminary effectiveness analysis

The presence or absence of PH will be determined using central review by blinded pediatric cardiologists of echocardiograms performed at the end of the study period. PH will also be graded as absent (normal), mild, moderate, or severe (Table 6). Results will be summarized by treatment assignment and dose group, and listed.

**Table 6:** Pulmonary hypertension composite score of echocardiographic measures

Subjective quantification of RV pressures	Absolute criteria	Supportive criteria		
	Septal geometry in systole	Shunt direction^**a**^	RV function^**b**^	RV/RA size^**c**^
Normal (< 1/3 systemic)	Septum round Eccentricity index^d^ ≤ 1.0	L to R	Normal	Normal
Mildly elevated (1/3–2/3 systemic)	Septal geometry mildly distorted, Eccentricity index 1.01–1.20	L to R	Normal	Mildly dilated
Moderately elevated (> 2/3 systemic)	Septum moderately distorted but not flat, Eccentricity index 1.21–1.4	L to R	Mildly depressed	Mod. dilated
Severely elevated (≥ systemic)	Septum flat or bowing into left ventricle, Eccentricity index > 1.4	R to L	≥ Mod. depressed	Severely dilated

^a^Across a PFO, ASD or PDA

^b^Measures of RV function to include: subjective estimates, RV fractional area change, tricuspid annular plane systolic excursion, RV strain and strain rate, Myocardial performance index

^c^RV to RA size based on subjective appearance

^d^Left ventricular (LV) eccentricity index (LVEI) = LV antero-posterior diameter / LV septo-lateral diameter

*Abbreviations*: *RV* right ventricular, *RA* right atrial, *PFO* patent foramen ovale, *ASD* atrial septal defect, *PDA* patent ductus arteriosus, *LV* left ventricular, *LVEI* left ventricular eccentricity index

Reference: McCrary AW, Barker PCA, Torok RD, Spears TG, Li JS, Hornik CP, et al. agreement of an echocardiogram-based diagnosis of pulmonary hypertension in infants at risk for bronchopulmonary dysplasia among masked reviewers. J Perinatol. 2019;39 (2):248–55

Risk and odds of PH in participants administered sildenafil versus placebo will be calculated (Risk Ratio, Odds Ratio [OR]), along with 95% confidence intervals (CI), overall by treatment group and for each assigned sildenafil dose. Logistic regression models will be used to determine association between baseline PH risk and odds of developing PH for participants administered sildenafil versus placebo. Additional potential confounders may be included in the models if data allow, such as GA, PNA, sex, respiratory support, events of special interest, medications of special interest, and comorbidities. Repeated measures may be used to allow for inclusion of random effects for site and siblings if required by intraclass correlation coefficient.

### Discussion

Despite the lack of safety and efficacy data in infants, neonatologists are increasingly using sildenafil to treat PH in premature infants. A review of data from the Pediatrix Medical Group Clinical Data Warehouse, a real-world data source representing approximately 20% of US NICU admissions, found an exponential increase in sildenafil use by > 1000%, second highest of all drugs from 2005 to 2010 [13]. Of the infants treated with sildenafil, 79% had BPD and 84% had PH. Two case reports documented the successful resolution of PH, measured by echocardiogram, in premature infants treated with sildenafil [14, 15]. In a small case series, enteral sildenafil in 25 premature infants with lung disease resulted in improved hemodynamics by echocardiogram [16]. A retrospective case series of 23 preterm infants treated with enteral sildenafil found a substantial improvement in BPD-associated PH based on echocardiography in 71% of patients for whom echocardiographic data were complete (15/21) [17]. These data confirm that neonatologists usually treat PH in infants with BPD and are likely unwilling to randomize to placebo; thus, a prevention approach is needed. This clinical trial will be essential to better understand the safety of this already commonly used medication in critically ill infants with severe BPD.

The strengths of this trial include a study design that will allow us to characterize the safety of sildenafil, confirm pharmacokinetics, and to explore the preliminary effectiveness and exposure-response relationship of sildenafil in the treatment of BPD-associated PH using echocardiography. Additionally, the unique R61/R33 funding mechanism of the grant affords 2 years of funding for trial planning and site start-up, followed by 3 years of enrollment. This additional time allows the investigators to make the study better quality by conducting a community engagement studio, developing modeling for adjusted analyses of efficacy, and focusing on site engagement. A corresponding study to develop a prediction tool for PH in infants with BPD using machine learning is currently in process, and will compound the value that this trial provides. Furthermore, the global rank scores gathered in the tertiary outcome of the trial will provide valuable data for future studies and insight into the morbidity associated with BPD, PH, and prematurity.

The strategies utilized in this study are innovative to the field of infant clinical research, and create value for both patients and the community. We have utilized community engagement studios to optimize recruitment and retention, and will use electronic health records (EHR) and electronic consent procedures to increase efficiency in conducting a study of a rare disease in a vulnerable population. This clinical trial will make significant strides in understanding the use of sildenafil in premature infants with PH, and provide a foundation for further exploration of utilization of sildenafil as a treatment modality for this condition.

Sildenafil is a promising potential intervention to prevent the development of PH in premature infants with BPD. Clinical trials of sildenafil specifically designed for premature infants are urgently needed. The current study will make significant contributions to scientific knowledge and safety of sildenafil in premature infants at risk for PH. Results from the study will be used by investigators to inform the design of a pivotal phase III efficacy trial.

## Supplementary Information

**Additional file 1: Appendix Table 1.** Schedule of Events.

**Additional file 2: Appendix Table 2.** Global Rank Endpoint.

## Data Availability

Following completion of the study, the investigator may publish the results of this research in a scientific journal. All public presentations (abstracts, manuscripts, slides and text of oral or other presentations, and text of any transmission through any electronic media) by participating investigators, participating institutions, and DCC, will be reviewed by the Publication Committee per the Publication Committee charter. All investigators funded by the NIH must submit or have submitted for them to the National Library of Medicine’s PubMed Central an electronic version of their final, peer -reviewed manuscripts upon acceptance for publication, to be made publicly available no later than 12 months after the official date of publication. The study is a clinical trial and will comply with the NIH policy that establishes the expectation that all investigators conducting clinical trials funded in whole or in part by the NIH will ensure that these trials are registered at ClinicalTrials.gov, and that results of these trials are submitted to ClinicalTrials.gov.

## Abbreviations

ALT: Alanine Aminotransferase
AST: Aspartate Aminotransferase
BPD: Bronchopulmonary Dysplasia
CI: Confidence Interval
CPAP: Continuous Positive Airway Pressure
DCRI: Duke Clinical Research Institute
DMS: Desmethyl Sildenafil
DSMB: Data Safety Monitoring Board
EHR: Electronic Health Records
ESI: Events of Special Interest
FDA: Food and Drug Administration
FiO_2_: Fraction of Inspired Oxygen
GA: Gestational Age
GEE: Generalized Estimating Equations
IRB: Institutional Review Board
IV: Intravenous
LPM: Liters per Minute
MAP: Mean Arterial Pressure
MedDRA: Medical Dictionary for Regulatory Activities
NAVA: Neurally Adjusted Ventilatory Assist
NCPAP: Nasal Continuous Positive Airway Pressure
NHLBI: National Heart Lung and Blood Institute
NICHD: National Institute of Child Health and Human Development
NICU: Neonatal Intensive Care Unit
NIPPV: Nasal Intermittent Positive Pressure Ventilation
OR: Odds Ratio
PD: Pharmacodynamics
PDA: Patent Ductus Arteriosus
PH: Pulmonary Hypertension
PK: Pharmacokinetics
PMA: Postmenstrual Age (gestational age plus postnatal age)
ROP: Retinopathy of Prematurity
SAE: Serious Adverse Event
U/L: Units per Liter
VEGF: Vascular Endothelial Growth Factor
WHO: World Health Organization
