# Supplementary material for: Safety of sildenafil in premature infants with severe bronchopulmonary dysplasia (SILDI-SAFE): a multicenter, randomized, placebo-controlled, sequential dose-escalating, double-masked, safety study
Source: BMC Pediatr. 2020 Dec 14;20:559. doi: 10.1186/s12887-020-02453-7 (PMC7735412; doi:10.1186/s12887-020-02453-7)
Supplement: Supplementary file 1 — Additional file 1: Appendix Table 1. Schedule of Events. [file 12887_2020_2453_MOESM1_ESM.docx]

Appendix Table 1: Schedule of Events

|  | **Screen/ Baseline** | **Treatment** | **Weaning^1^**  **Cohort 2 and 3** | **Follow-up** | **Final study assessment** |
| --- | --- | --- | --- | --- | --- |
| **Time (Day)** | Predose^2^ | 1‑28^3^  (± 1 Day) | Weaning  Day 1-6 | Day 1-28 post last study drug dose | Discharge or Transfer |
| Informed consent | x |  |  |  |  |
| Randomization^4^ | X |  |  |  |  |
| Demographics^5^ | X |  |  |  |  |
| Physical examination | x |  |  | X (weekly) | X |
| Medical history | x |  |  |  |  |
| Actual Weight | X | X (weekly) |  | X (weekly) | X |
| Mean arterial pressure | X | X | Last day of wean | X (weekly) |  |
| Respiratory assessment | x | X^6^ (daily) | X^6^ (daily) | x^6^ (daily) | X |
| Laboratory evaluations^7^ | X | x (weekly) |  | x (weekly) |  |
| Study drug administration |  | x^8^ | X |  |  |
| Concomitant medications | X | X |  |  |  |
| Concomitant medications of special interest^9^ | X | X | X | X | X |
| Adverse events (including SAEs and ESIs)^10^ | X | X | X | X |  |
| Global Rank |  |  |  |  | X |
| Echocardiogram | x | X^11^ | x^11^ | X | X^11^ |
| Cardiac catheterization reports^11^ | X | X | X | X | X |
| PK sampling |  | X (after Day 7) |  |  |  |
| Discharge information, including ROP^12^ |  |  |  |  | x |

Abbreviations: AE, adverse event; PK, pharmacokinetics; PMA, postmenstrual age; ROP, retinopathy of prematurity; SAE, serious adverse event

^1^ Weaning for Cohort 2 and 3 will begin following Day 28 or following the last day of study drug if participant was withdrawn from study drug prior to Day 28 and the dose was escalated to ≥ 0.5 mg/kg IV or ≥ 1 mg/kg enteral.

^2^ Refers to < 24 hours (except laboratory evaluations) and < 14 days (for echocardiogram) prior to start of study drug that these procedures may be conducted and may be the same calendar date as Day 1; informed consent may be obtained any time prior to the initiation of any study procedures.

^3^ Collect all safety follow-up and remaining assessments if early withdrawal of study drug occurs during the 28‑day treatment period. AE and SAE follow‑up is through 28 days post last dose of study drug.

^4^ Randomization will occur on Day -1 (1 day prior to first day of treatment).

^5^ Participant demographics (including birthweight, gestational age at birth) and maternal race/ethnicity will be collected.

^6^ Recorded daily during the treatment, weaning, and follow-up periods.

^7^ If not performed per standard of care, laboratory evaluations must be performed within 72 hours prior to randomization and weekly during treatment and weekly during the first 14 days of the follow-up period.

^8^ Dosing may be adjusted according to any weight changes determined at baseline, study Days 7, 14, and 21.

^9^ Concomitant medications of special interest will be recorded during the entire study period.

^10^ AEs will be collected following initial study-specific procedure (e.g., screening blood draws, study drug dosing) through 28 days post last dose of study drug.

^11^ If performed per local standard of care

^12^ Transfer, discharge, duration of hospitalization or death; record if treatment for ROP was required.
