# Supplementary material for: Safety of sildenafil in premature infants with severe bronchopulmonary dysplasia (SILDI-SAFE): a multicenter, randomized, placebo-controlled, sequential dose-escalating, double-masked, safety study
Source: BMC Pediatr. 2020 Dec 14;20:559. doi: 10.1186/s12887-020-02453-7 (PMC7735412; doi:10.1186/s12887-020-02453-7)
Supplement: Supplementary file 2 — Additional file 2: Appendix Table 2. Global Rank Endpoint. [file 12887_2020_2453_MOESM2_ESM.docx]

**Appendix Table 2. Global Rank Endpoint**

| Rank | Description |
| --- | --- |
| 1 | Mortality |
| 2 | *Neurodevelopmental impairment (NDI): N/A* |
| 3 | Extracorporeal life support (ECLS; i.e., “ECMO”) |
| 4 | Tracheostomy |
| 5 | Periventricular leukomalacia (PVL) |
| 6 | *Blindness: N/A* |
| 7 | *Deafness: N/A* |
| 8 | *Hypoxic ischemic encephalopathy (HIE): N/A* |
| 9 | Seizures |
| 10 | Dialysis |
| 11 | Retinopathy of prematurity (ROP) |
| 12 | Short gut syndrome/intestinal failure |
| 13 | *Bronchopulmonary dysplasia (BPD): N/A* |
| 14 | Ventriculoperitoneal (VP) shunt |
| 15 | Gastrostomy tube |
| 16 | Surgical necrotizing enterocolitis/intestinal perforation (NEC/IP) |
| 17 | Patent ductus arteriosus (PDA) |
| 18 | Meningitis |
| 19 | Sepsis |
| 20 | Duration of hospitalization |

The following definitions and data handling conventions will be adopted:

1. Mortality: Death during primary hospitalization
2. NDI: N/A
3. ECLS (ECMO): receipt of extracorporeal life support (or extracorporeal membranous oxygenation)
4. Tracheostomy: receipt of tracheostomy for the purposes of continued mechanical ventilation or positive airway pressure. Tracheostomy for airway anomalies (e.g. subglottic stenosis) without mechanical ventilation or positive airway pressure will be recorded but will not count for the purposes of the GRS
5. Periventricular leukomalacia (PVL): echolucency (including porencephalic cyst) on cranial ultrasound/MRI/CT obtained between 36-44 weeks postmenstrual age; or “periventricular leukomalacia” or “PVL” is a diagnosis in the medical chart
6. Blindness— N/A
7. Deafness—bilateral: N/A
8. Hypoxic ischemic encephalopathy (HIE)—N/A
9. Seizures: diagnosis of seizures and receipt of an anti-epileptic/anti-seizure medication
10. Dialysis: receipt of peritoneal dialysis or other renal replacement therapy (i.e., hemodialysis or continuous renal replacement therapy); Acute dialysis is for <3 months or <90 days; chronic dialysis is 3 months (90 days) or greater.
11. Retinopathy of prematurity: receipt of intravitreal injection of medications to prevent ROP or cryotherapy or laser photocoagulation or other ophthalmic surgery to correct ROP (i.e. scleral buckle/vitrectomy)
12. Short gut syndrome/intestinal failure: total parenteral nutrition (TPN) dependent at discharge or bowel transplant
13. Bronchopulmonary dysplasia (BPD): N/A
14. Ventriculoperitoneal shunt: receipt of ventriculoperitoneal shunt, subgaleal shunt, or other cerebrospinal fluid diversion
15. Gastrostomy tube: receipt of a gastrostomy tube for enteral feedings
16. Surgical necrotizing enterocolitis/intestinal perforation (NEC/IP): receipt of a laparotomy or drain for NEC or IP
17. Patent ductus arteriosus (PDA): receipt of PDA ligation or cardiac catheterization for the purposes of closing the PDA
18. Meningitis/encephalitis: positive cerebrospinal fluid culture or CSF parameters suggestive meningitis not considered a contaminant
19. Sepsis: positive blood culture of an organism not considered a contaminant
20. Duration of hospitalization: Post menstrual age (PMA) at discharge to home (not transfer to another facility): 36-39 weeks PMA, 40-43 weeks PMA, 44-47 weeks PMA, 48-51 weeks PMA, ≥52 weeks PMA
